# Differentiating Primary and Recurrent Lesions in Patients with a History of Breast Cancer: A Comprehensive Review

**DOI:** 10.31661/gmj.v13i.3340

**Published:** 2024-04-22

**Authors:** Anita Zarghami, Seyed Abbas Mirmalek

**Affiliations:** ^1^ Department of Surgery, Shahid Beheshti University of Medical Sciences, Tehran, Iran; ^2^ Department of Surgery, Tehran Medical Sciences, Islamic Azad University, Tehran, Iran

**Keywords:** Breast Cancer, Recurrence, Mastectomy, Lumpectomy, Mammography

## Abstract

Breast cancer (BC) recurrence remains a concerning issue, requiring accurate identification and differentiation from primary lesions for optimal patient management. This comprehensive review aims to summarize and evaluate the current evidence on methods to distinguish primary breast tumors from recurrent lesions in patients with a history of BC. Also, we provide a comprehensive understanding of the different imaging techniques, including mammography, ultrasound, magnetic resonance imaging, and positron emission tomography, highlighting their diagnostic accuracy, limitations, and potential integration. In addition, the role of various biopsy modalities and molecular markers was explored. Furthermore, the potential role of liquid biopsy, circulating tumor cells, and circulating tumor DNA in differentiating between primary and recurrent BC was emphasized. Finally, it addresses emerging diagnostic modalities, such as radiomic analysis and artificial intelligence, which show promising potential in enhancing diagnostic accuracy. Through comprehensive analysis and review of the available literature, the current study provides an up-to-date understanding of the current state of knowledge, challenges, and future directions in accurately distinguishing between primary and recurrent breast lesions in patients with a history of BC.

## Introduction

Breast cancer (BC) is one of the most common types of cancer affecting women worldwide [1]. It is characterized by the growth of abnormal breast cells, often forming a lump or mass. The diagnosis and management of BC can be complex, particularly when distinguishing between primary and recurrent lesions [2].

Primary lesions refer to the initial BC diagnosis, where the tumor originates and grows in the breast tissue [3]. Recurrent lesions, on the other hand, occur when BC returns after the completion of treatment [4]. This recurrence can manifest as a local recurrence in the breast or chest wall, regional recurrence in the lymph nodes, or as distant metastases in distant organs such as the bones, lungs, or liver [5]. Hence, distinguishing between primary and recurrent lesions is crucial in the management of patients with a history of BC as it impacts treatment decisions and prognosis. Indeed, primary lesions are often managed with curative intent, whereas recurrent lesions may require a more aggressive or palliative approach [6].

Several factors help differentiate primary and recurrent lesions in patients with BC. The first step is to evaluate the patient’s medical history, including the type and stage of the initial BC, treatment modalities received, and any surveillance imaging or follow-up conducted [7]. A thorough physical examination is also essential to assess the lesion’s location, size, and characteristics [8].

In addition to medical history and clinical examination, imaging plays a vital role in distinguishing between primary and recurrent lesions [8][9]. Imaging techniques such as mammography, ultrasound, and magnetic resonance imaging (MRI) are commonly used. Mammography provides detailed images of the breast tissue and can often identify abnormalities such as masses, calcifications, or architectural distortions [10]. Ultrasound can help differentiate between solid and cystic lesions and determine their features, such as irregular margins or vascularity [11]. MRI, with its superior soft tissue contrast, can help detect small lesions and assess the extent of involvement [12].

However, imaging alone may not differentiate between primary and recurrent lesions. Biopsy involves obtaining a sample of the suspicious tissue and analyzing it under a microscope. This helps determine the presence of cancer cells, the histological subtype, and whether the lesion represents a recurrence or a new primary tumor [13]. Biopsy techniques can vary, including fine needle aspiration (FNA), core needle biopsy (CNB), or surgical biopsy, depending on the size, location, and accessibility of the lesion [14]. Also, the differentiation between primary and recurrent lesions is necessary for appropriate treatment planning [15]. Primary lesions are often managed with a multimodal approach, including surgery, chemotherapy, radiation therapy, and targeted therapies based on tumor characteristics [16]. In contrast, the management of recurrent lesions may involve a combination of surgery, radiation therapy, systemic therapies, and palliative care [17]. Hence, in this review, we aimed to highlight the challenges and importance of differentiating between primary and recurrent lesions in patients with a history of BC. Also, we overview identification techniques, proper treatments, prognosis estimation, and patient outcomes.

## Clinical Presentations and Assessments

Symptoms and Complaints

Patients with a history of BC may experience primary and recurrent lesions during their treatment procedure [18]. Symptoms and complaints associated with these lesions can differ depending on various factors, such as the cancer stage, the type of treatment received, and individual patient characteristics [19]. Symptoms and complaints associated with primary lesions may include the presence of a lump or thickening in the breast or underarm area, changes in breast size or shape, skin dimpling or puckering, nipple retraction or inversion, nipple discharge, and persistent breast pain [20]. These symptoms often prompt patients to seek medical attention, leading to BC diagnosis.

On the other hand, recurrent lesions occur when BC returns after a period of remission or successful treatment. Common symptoms of recurrent lesions can be similar to primary lesions, although they may vary in intensity or presentation [21]. Patients may notice the reappearance of a lump or mass, changes in breast appearance, skin abnormalities, nipple changes, or pain [22]. Recurrent lesions can be associated with physical and emotional distress, as patients may have already gone through previous treatments and experienced the impact of the disease on their lives [21][22].

Patients with a history of BC need to be aware of any new symptoms or changes in their breast tissue. It is worth noting that not all symptoms or complaints are indicative of cancer recurrence, as various benign conditions can also produce similar signs [23][24]. Nevertheless, it is always recommended to consult with a medical professional to assess any concerns related to primary or recurrent breast lesions in patients with a history of BC. Through ongoing monitoring and personalized care, healthcare teams can provide appropriate interventions and support to help manage symptoms and improve patients’ quality of life (QoL) [25].

Physical Examination

Physical examination is an essential component of the comprehensive evaluation of patients with a history of BC, both for primary and recurrent lesions. The examination aims to assess the size, location, and characteristics of any existing lesions, evaluate the involvement of neighboring structures, and identify any signs of disease recurrence [26]. Typically, it starts by inspecting the breasts for any visible abnormalities. This may involve comparing the size, shape, and symmetry of the breasts, looking for changes in the skin’s color and/or texture, and evaluating the nipple and areola for any signs of retraction or discharge [27]. Additionally, the physician palpates the breasts carefully, feeling for any lumps, nodules, or skin thickening.

In the cases where a primary breast lesion is detected, the examination should focus on assessing its characteristics, including the size, shape, and mobility of lesions, as well as evaluating its consistency, tenderness, or fixation to the underlying tissues [28]. The physician may also assess regional lymph nodes, including those in the axilla and supraclavicular areas, to determine if the cancer has spread [26].

Physical examinations are necessary for patients with a history of BC and presenting with recurrent lesions. Indeed, the physician should carefully evaluate the site and size of the recurrence, comparing it to previous images or notes to determine if there has been any growth or changes in appearance [27]. Also, they assess the surrounding tissues and lymph nodes to check for any signs of metastasis or spread of the disease [27].

In addition to a thorough breast examination, the physician may perform a systemic evaluation to assess the patient’s overall health and well-being [28]. This may involve checking vital signs, evaluating patient’s general appearance and nutritional status, and conducting a general physical examination to ensure no signs of systemic disease or complications [28]. Overall, by using a comprehensive evaluation of the breasts, lymph nodes, and surrounding structures, physicians can collect valuable data to guide further diagnostic testing, treatment planning, and patient management.

Assessment of Lymph Nodes

Lymph nodes play a critical role in the spread of cancer cells, as they can serve as a pathway for metastasis to other body parts [29]. Thus, a thorough examination of the lymph nodes is essential for determining the extent and stage of the disease. Also, lymph node involvement is a prognostic factor and significantly impacts treatment decisions [30]. Typically, a combination of clinical examination, imaging techniques such as ultrasound or MRI, and a sentinel lymph node biopsy may be performed to assess the lymph nodes [31].

In cases where recurrent BC is suspected, a similar approach is taken to assess the lymph nodes. This recurrence can occur in the breast tissue, the chest walls, or the nearby lymph nodes [32]. In addition to clinical examination and imaging methods, a biopsy of the recurrent lesion or lymph nodes may be necessary to confirm the presence of cancer cells [32].

Different techniques are used to assess lymph nodes, depending on the level of suspicion or the individual patient’s characteristics. For instance, a sentinel lymph node biopsy involves the identification and removal of the first lymph node(s) that cancer cells are likely to spread to from the primary tumor site [33]. This selective approach minimizes the removal of unnecessary lymph nodes, reducing the risk of lymphedema and other complications [34].

Furthermore, technological advancements have improved the assessment of lymph nodes in patients with a history of BC. In recent years, new imaging techniques such as positron emission tomography-computed tomography (PET-CT) scans and molecular imaging utilizing radiolabeled tracers have emerged as promising diagnostic tools [35][36]. These methods allow for a more accurate assessment of lymph node involvement and assist in treatment planning decisions.

## Diagnostic Imaging Modalities

Mammography

Mammograms are highly effective in detecting breast abnormalities and changes that may indicate the presence of a tumor [37]. When a patient with a history of BC undergoes a mammogram, the radiologist carefully compares the current imaging results with previous mammograms. This comparison allows them to evaluate any changes in the breast tissue over time [38]. If a new lesion is found, it can indicate the presence of a primary tumor rather than a recurrent lesion [39].

In addition to comparing previous mammograms, it helps determine the characteristics of the detected lesions. Differentiating between primary and recurrent lesions is essential because the management strategies for both types may differ significantly [40]. Mammography enables radiologists to assess various factors, such as the lesions’ size, shape, and margins, which help differentiate between the two [41]. For example, primary lesions may appear as distinct masses, while recurrent lesions may exhibit irregular borders or be associated with calcifications [42].

Furthermore, mammography can be used to guide additional diagnostic procedures, such as ultrasonography or biopsy, which can provide further clarity in differentiating primary and recurrent lesions [43]. These additional tests can help assess the lesion’s characteristics, such as its cellular composition, which aids in determining the origin of the lesion [44].

Ultrasound

This imaging modality uses high-frequency sound waves to produce detailed images of the breast tissue, allowing radiologists to evaluate suspicious areas and determine their nature [45]. Ultrasound could distinguish primary and recurrent cancer in patients with new breast lesions [46]. By examining the characteristics of the lesion, such as its shape, margins, and internal echo pattern, radiologists can make an initial assessment [45]. Primary cancers often exhibit irregular shapes, ill-defined margins, and heterogeneous internal echoes, while recurrent lesions may have more regular shapes and well-defined margins [46].

Furthermore, ultrasound can aid in distinguishing between solid masses and fluid-filled cysts. Cysts-common lesions in the breast-appear as well-defined and fluid-filled structures on ultrasound and are typically benign without a major concern [47]. On the other hand, solid masses may indicate a possible tumor, either primary or recurrent [48]. The radiologist carefully evaluates the characteristics of the solid mass, including its size, shape, and presence of blood flow, to determine its likelihood of malignancy [49].

In addition, ultrasound-guided biopsies can be performed to obtain samples from suspicious areas for further analysis [50]. This minimally invasive procedure targets specific areas of concern and helps distinguish between benign and malignant lesions [51]. Ultrasound-guided biopsies can target the lesion by visualizing the biopsy needle in real time, ensuring an accurate diagnosis and appropriate treatment planning [52].

MRI

Patients with BC often require long-term surveillance to detect any recurrence or new lesions [7]. MRI provides a non-invasive method to examine breast tissue and assess any changes that may indicate cancerous or non-cancerous growth [53].

Also, it offers a more comprehensive evaluation than other imaging techniques, such as mammography or ultrasound [45]. One of the key advantages of MRI is its ability to detect small lesions that may be missed by other imaging modalities [54]. Indeed, the high sensitivity of MRI allows for detecting lesions as small as a few millimeters, which is vital for early diagnosis and treatment [55]. Additionally, the multiplanar imaging provided by MRI allows for accurate assessment of the lesion’s size, location, and extent, aiding treatment planning [54]. MRI is beneficial in cases where the initial diagnosis reveals an ambiguous or inconclusive lesion [53]. Furthermore, in patients who have undergone breast-conserving surgery or mastectomy, MRI helps assess the effectiveness of the surgery and detect any residual or recurrent lesions [56][57].

PET-CT

Nuclear medicine imaging, specifically PET-CT, plays a significant role in differentiating primary and recurrent lesions in patients with a history of BC. This advanced imaging technique combines the benefits of both PET and CT scans to provide detailed and accurate information about the location, size, and metabolic activity of lesions within the body [58].

Indeed, PET/CT scans use an injected radioactive tracer, usually fluorodeoxyglucose (FDG) [59]. FDG is taken up by actively dividing cancer cells, making it possible to detect areas of increased metabolic activity [58]. Additionally, the CT component of the scan provides detailed anatomical information, allowing for precise localization of the lesions seen on the PET scan [58][60]. Actually, traditional imaging techniques, such as mammography or ultrasound, mainly rely on anatomical changes and may miss small recurrent lesions [60]. PET/CT, on the other hand, assesses metabolic activity, which is often increased in recurrent tumors, even when anatomical changes are minimal [60][61]. Moreover, PET/CT imaging is valuable in cases where there is suspicion of distant metastasis or involvement of multiple sites [62]. By providing a whole-body scan, PET/CT could detect the presence of additional lesions, helping to determine the extent of disease involvement within the body [63].

Optical Imaging Techniques

These techniques applied non-invasive methods to provide high-resolution images of tissue structures, allowing for early detection and accurate diagnosis of tumoral lesions [64].

One important optical imaging technique is optical coherence tomography (OCT), which uses light waves to create cross-sectional images of tissues [65]. In BC, OCT can help distinguish between primary and recurrent lesions by assessing the thickness and density of tissue layers [65]. Also, it could provide information about the presence of blood vessels and the extent of tumor infiltration into surrounding tissues [66]. By visualizing the microstructure of breast tissues, OCT enables clinicians to identify subtle changes in cellular architecture, effectively differentiating between primary and recurrent tumors [65].

Diffuse optical spectroscopy (DOS) is another technique that applies near-infrared light to measure the absorption and scattering properties of tissues [67]. DOS can detect malignancy-related changes by analyzing breast tissue’s biochemical composition and oxygenation levels [68]. This technique can also assess angiogenesis as a key feature of recurrent lesions [67]. By quantifying these parameters, DOS enhances the accuracy of differentiating between primary and recurrent BC lesions [69].

Furthermore, multiphoton imaging is another optical technique that uses high-intensity laser pulses to excite fluorescent molecules within tissues, allowing for the visualization of cellular and molecular changes [70]. Multiphoton imaging could reveal alterations in cellular metabolism, tissue architecture, and collagen composition indicative of cancer progression [71].

## Pathological Evaluation

FNA

FNA is a minimally invasive procedure that allows for collecting cells or fluid from a suspicious breast lesion for diagnostic evaluation [72]. One of the main benefits of FNA is its ability to differentiate between a primary tumor and a recurrence [73]. FNA provides cytological samples that can be examined under a microscope to determine the presence of cancer cells. By comparing these cells with the patient’s previous cancer pathology report, pathologists can determine whether the lesion indicates a recurrent tumor or a new primary tumor [73].

Another advantage of FNA is its ability to assess the characteristics of the lesion, such as its size and tumor grade [72]. Also, pathologists can determine the level of cellular atypia, nuclear features, and mitotic activity [73]. Hence, these features aid in determining the aggressiveness of the tumor and help in guiding further treatment decisions [74]. Additionally, FNA can also aid in the detection of distant metastasis [75]. Indeed, FNA can be performed on suspicious nodules in other organs (e.g., lungs) to ascertain whether the lesion represents metastasis from the primary BC [76].

CNB

This procedure involves the extraction of tissue samples from suspicious areas in the breast using a large needle, which is then examined under a microscope [77]. CNB provides accurate information about the characteristics of the lesion, aiding in the differentiation between primary and recurrent tumors [78].

CNB could provide histological confirmation and help pathologists identify specific cancer markers and determine the nature of the lesion [78]. Furthermore, CNB allows for the assessment of the histological grade of the lesion, providing invaluable information regarding its aggressiveness and potential for metastasis [79]. Also, CNB enables the analysis of biomarkers, such as hormone receptors and human epidermal growth factor receptor 2 (HER2), which play a critical role in guiding targeted therapy decisions [80]. This is particularly important when a patient presents with a suspicious mass after treatment [78].

Surgical Biopsy

The primary purpose of a surgical biopsy is to obtain a tissue sample from the suspicious lesion for histopathological analysis [81]. Indeed, pathologists can differentiate primary tumors from recurrent lesions using various histological features such as tumor grade, hormone receptor status, HER2/neu expression, and genetic mutations [82].

Also, they can evaluate different subtypes of BC, e.g., invasive ductal carcinoma and invasive lobular carcinoma, that exhibit different growth patterns and cellular arrangements [83].

Another crucial aspect in differentiating between primary and recurrent lesions is the presence or absence of specific biomarkers. Hormone receptor status, such as estrogen receptor (ER) and progesterone receptor (PR) expression, can provide valuable information about the hormone dependency of the tumor [84]. Additionally, the overexpression or amplification of the HER2/neu gene is an important biomarker for the diagnosis and treatment of BC [85]. By assessing these biomarkers through immunohistochemistry (IHC) or fluorescence in situ hybridization (FISH) analysis on the tissue sample obtained from surgical biopsy, oncologists can determine if the lesion is consistent with a primary tumor or a recurrence [86]. Furthermore, genetic mutations, such as breast cancer 1 (BRCA1) and BRCA2 mutations, can also help differentiate primary from recurrent lesions [87].

## Molecular and Genomic Profiling

IHC

One key application of IHC is detecting hormone receptor status, specifically ER and PR [86]. These receptors play an important role in BC development and progression. If the primary tumor was ER and/or PR positive, assessing the receptor status in the recurrent lesion can provide valuable information for treatment decision-making [87].

Another significant role of IHC in differentiating primary and recurrent lesions is the detection of HER2 overexpression [80][85]. HER2-positive BC is characterized by aggressive tumor behavior and requires targeted therapies such as trastuzumab or pertuzumab [88]. Hence, using IHC, pathologists could accurately evaluate HER2 protein expression levels in the recurrent lesions to establish their molecular subtype and differentiate them from primary tumors [89].

Furthermore, IHC could identify specific tumor markers, such as Ki-67, which indicates the proliferative activity of cancer cells [90]. Ki-67 expression levels can vary between primary and recurrent lesions, providing valuable information about tumor aggressiveness and assessing treatment response [91]. Indeed, higher Ki-67 expression in recurrent lesions compared to primary tumors suggests more aggressive tumor behavior and may influence treatment approaches, such as chemotherapy or targeted therapies [92].

FISH

FISH is a molecular diagnostic technique that can detect and visualize specific genetic changes or abnormalities in the cells [93]. For example, FISH allows for the identification of HER2 gene amplification by fluorescently labeled probes that detect HER2 signals in tumor cells [94].

Also, FISH helps detect alterations in the ER and PR genes and determines their copy numbers, which aids in distinguishing between primary and recurrent lesions [95]. This information assists clinicians in personalizing treatment strategies and predicting the likelihood of response to hormonal therapy.

Furthermore, FISH can detect chromosomal abnormalities, such as deletions or rearrangements, that indicate BC progression [96]. The analysis of specific chromosomal regions through FISH can identify tumor-associated genetic alterations, which lead to determining the clonal relationship between primary and recurrent lesions [97]. Indeed, if the chromosomal changes are similar, it suggests that the recurrent lesion originates from the primary tumor, whereas different changes indicate a new primary cancer.

Next-Generation Sequencing (NGS)

NGS technology allows for a comprehensive analysis of the genetic and molecular characteristics of tumors [98]. Indeed, by sequencing the DNA or RNA from a patient’s tumor sample, NGS provides a high-resolution view of the tumor’s genomic landscape [99]. This enables the identification of specific genetic alterations, such as mutations, amplifications, or deletions, which can distinguish between primary and recurrent lesions. Furthermore, NGS-based approaches can help identify potential therapeutic targets in recurrent BC cases [100].

Liquid Biopsies

Liquid biopsies have gained significant attention in recent years due to their potential to revolutionize cancer diagnostics, particularly in patients with a history of BC [101]. Liquid biopsies offer a non-invasive alternative to traditional tissue biopsies, which require an invasive procedure to collect samples from the primary or recurrent lesion [102].

Liquid biopsies, on the other hand, analyze circulating tumor cells (CTCs) and circulating tumor DNA (ctDNA) that are shed into the bloodstream by tumors [103]. Hence, liquid biopsy samples can be easily obtained through a simple blood draw, making them a more convenient and less risky option for patients [102].

Also, liquid biopsies can be crucial in monitoring disease recurrence and treatment response over time [104].

Regular monitoring of ctDNA levels in the bloodstream can help detect the presence of minimal residual disease (MRD), even before it is clinically evident [104]. Moreover, liquid biopsies enable the assessment of tumor heterogeneity, a phenomenon in which primary and recurrent lesions may harbor different genetic mutations [102][105].

## Emerging Technologies and Techniques

Radiomics and Machine Learning

Radiomics is the extraction of quantitative imaging features from medical images, such as mammograms or MRI scans [106]. These features capture the heterogeneity and characteristics of the tumor, providing valuable information for diagnosis and prognosis [107]. Machine learning algorithms then utilize these radiomics features to develop models that can accurately differentiate between primary and recurrent lesions [108].

One of the key advantages of using radiomics and machine learning is their ability to analyze large amounts of data in a systematic and objective manner. By extracting numerous imaging features, such as texture, shape, or intensity-based parameters, radiomics comprehensively assess the tumor’s characteristics [106].

Moreover, radiomics and machine learning provide a non-invasive and cost-effective approach to distinguish primary from recurrent lesions [107]. Indeed, radiomics-based analysis of medical images eliminates the need for these invasive procedures and reduces the potential risks and discomfort for patients [106].

CTCs

CTCs are cancer cells detached from the primary tumor and enter the bloodstream [103]. These cells can potentially spread to other body parts and form secondary tumors. Hence, by analyzing the presence and characteristics of CTCs, healthcare professionals can distinguish primary tumors from recurrent lesions [109].

Also, CTCs can act as a biomarker for tumor recurrence. Indeed, CTCs can be detected earlier than other diagnostic methods, such as imaging techniques, as they represent disseminated cancer cells even before macroscopic metastases occur [103][110]. Hence, tracking CTCs can aid in the early detection of recurrent lesions and enable timely intervention to improve patient outcomes. Additionally, the analysis of CTCs could reveal the mechanisms of treatment resistance [111]. Recurrent lesions often resist previously effective therapies, which poses a challenge in their management. By studying CTCs, researchers can uncover the genetic and molecular changes contributing to treatment resistance, facilitating the development of alternative treatment strategies [112].

ctDNA

The ctDNA refers to small fragments of tumor DNA that are released into the bloodstream by cancer cells [103]. By analyzing ctDNA and its specific mutations, researchers can obtain valuable insights into the genetic profile of a tumor, allowing for more accurate diagnosis and treatment decisions. One key role of ctDNA in differentiating primary and recurrent lesions is its ability to detect MRD [113].

After initial BC treatment, residual cancer cells are always likely to remain in the body. ctDNA analysis can detect these cells, providing information about the presence of MRD, which is important in identifying patients at higher risk of recurrence, as it allows for more tailored and proactive treatment strategies [114].

Traditional imaging techniques may not always be sufficient in distinguishing between local, regional, or distant recurrences [115]. However, ctDNA analysis can provide additional information about the genetic alterations associated with the site of recurrence [116]. This enables more accurate localization and appropriate treatment planning involving surgical intervention, radiation therapy, or systemic treatment modalities [115].

Furthermore, ctDNA analysis can potentially overcome the challenges associated with tissue biopsy and the heterogeneity of tumors [117].

Obtaining tumor tissue for genetic analysis can be invasive and may not always be feasible or safe, especially in recurrent cases. Additionally, due to the genetic heterogeneity of tumors, a single biopsy may not capture all mutations, leading to potential inaccuracies in treatment decisions [117]. ctDNA analysis, being non-invasive and capable of capturing the mutational landscape of cancer cells, provides a comprehensive and dynamic view of the tumor’s genetic profile, facilitating more informed decision-making [118].

Electrical Impedance Spectroscopy (EIS)

The EIS is a non-invasive technique that measures the electrical properties of tissues to differentiate primary and recurrent lesions in patients with a history of BC [119][120]. One of the primary advantages of using EIS is its ability to assess tissue heterogeneity, often indicative of cancerous growth [121]. The electrical properties of healthy breast tissue differ significantly from those of cancerous tissue. By analyzing impedance values at different frequencies, EIS can identify changes in tissue conductivity and permittivity, allowing for the differentiation between primary and recurrent lesions [120]. Moreover, EIS can help distinguish between benign and malignant lesions, reducing unnecessary biopsies and related healthcare costs for patients [122].

Another advantage of EIS is its non-invasive nature, which reduces patient discomfort and improves compliance [120]. Unlike traditional imaging techniques, such as mammography or biopsy, EIS does not involve exposure to ionizing radiation or the need for tissue sampling [121]. This makes EIS an attractive option for regular monitoring of patients with a history of BC, minimizing the risk of radiation-associated complications and patient anxiety.

## Challenges and Limitations

Patients with a history of BC often face the challenge of distinguishing between primary and recurrent lesions, which is critical for appropriate management and treatment decisions. Clinical challenges arise due to several factors, including the variability in presentation and the inherent limitations of diagnostic tests [123]. The presentation of recurrent lesions may vary, sometimes appearing as a new mass, changes in the surgical scar, or even as distant symptoms like bone pain or cough [124]. These nonspecific symptoms can make it challenging to attribute their cause to recurrent BC and lead to unnecessary investigations or delays in diagnosis. Local recurrences can present as ill-defined masses or architectural distortions, which can be difficult to distinguish from scars or benign changes [124]. Imaging findings alone may not provide sufficient evidence to differentiate between these lesions definitively. Indeed, one of the main limitations is the possibility of false-positive or false-negative results [125]. Another limitation is the difficulty in distinguishing between scar tissue and tumor recurrence in imaging studies [126]. So, scar tissue can cause distortion or architectural changes in the breast tissue, making it challenging to interpret imaging findings accurately.

The sensitivity of imaging modalities, such as mammography, ultrasound, and MRI, also varies depending on the size, location, and characteristics of the lesions [127]. Smaller lesions, especially those less than 1 cm in size, may be difficult to detect on mammograms or ultrasounds, leading to missed diagnoses. Similarly, recurrent lesions located deep within the breast tissue may be difficult to visualize on mammograms or ultrasounds due to their location [128]. Moreover, imaging techniques may not always be able to provide a definitive diagnosis and may require additional diagnostic procedures, such as biopsies, for confirmation.

## Multidisciplinary Approach to Diagnosis: Tumor Board Meetings

These meetings involve a multidisciplinary team of healthcare professionals, including medical oncologists, radiation oncologists, surgical oncologists, radiologists, pathologists, and sometimes genetic counselors [129]. The primary purpose of tumor board meetings is to collaboratively review patient cases and make evidence-based treatment decisions [130]. Indeed, tumor board meetings allow all involved specialists to discuss and analyze a patient’s medical history, imaging results, pathology reports, and other relevant diagnostic information. Through this comprehensive evaluation, the tumor board can collectively understand the patient’s case and determine the most accurate diagnosis.

In addition to aiding in the differentiation of primary and recurrent lesions, tumor board meetings also contribute to developing personalized treatment strategies for patients with BC [131]. The multidisciplinary nature of these meetings allows for a comprehensive discussion of the patient’s case, considering factors such as tumor stage, molecular subtype, previous treatments, potential hereditary factors, and the patient’s overall health status. By pooling the expertise of various specialists, tumor board meetings can help tailor treatment plans that offer the highest chances of success while minimizing side effects and preserving the patient’s QoL [132].

## Management Considerations Based on Lesion Differentiation

Surgical Interventions

For patients with primary BC, surgical removal of the tumor is often the initial step in their treatment plan [133]. The type of surgery depends on various factors, such as the tumor size and location, the cancer stage, and the patient’s personal preferences.

One common surgical intervention for primary BC is a lumpectomy or breast-conserving surgery [134]. During this procedure, the surgeon removes only the tumor and a small margin of healthy tissue surrounding it while preserving the rest of the breast. This approach aims to achieve complete tumor removal while allowing for cosmetically pleasing results [135]. Following a lumpectomy, patients may undergo radiation therapy to reduce further the risk of cancer recurrence in the affected breast [134].

Alternatively, some patients may opt for a mastectomy, which involves the complete removal of the breast tissue [136]. A total mastectomy removes all breast tissue, while a modified radical mastectomy includes the removal of breast tissue along with the adjacent lymph nodes. In cases where the cancer has spread extensively or the patient carries specific genetic mutations, a prophylactic bilateral mastectomy may be recommended to reduce the risk of future BC development [137].

Surgical interventions in patients with recurrent BC aim to remove the recurrent lesions and alleviate symptoms [138]. Sometimes, a second lumpectomy may be possible when the recurrence is localized and the patient has previously undergone breast-conserving surgery. However, for patients with a more extensive and/or history of lumpectomy, a mastectomy may be considered the surgical intervention of choice [139]. In addition to surgical removal of recurrent lesions, the management of recurrent BC often involves a multidisciplinary approach, including adjuvant therapies such as chemotherapy, radiation therapy, and hormone therapy [138]. These treatments aim to eliminate any residual cancer cells, reduce the risk of further recurrences, and improve overall survival (OS).It is important to note that surgical interventions for primary and recurrent BC should be individualized to each patient’s specific circumstances and preferences. The treatment plan is often decided through a shared decision-making process between the patient, surgeon, and oncology team, considering factors such as the extent of the disease, overall health status, and the patient’s desire for breast preservation [140]. The ultimate goal of surgical interventions in patients with a history of BC is to provide optimal cancer control while ensuring the best possible cosmetic and psychosocial outcomes [141].

Radiation Therapy

It is commonly used after surgery, such as lumpectomy or mastectomy, to eliminate any remaining cancer cells, decrease the risk of local recurrence, and improve OS rates [142]. For primary lesions, radiation therapy is usually administered after breast-conserving surgery. By delivering high-energy radiation directly to the affected site, radiation therapy can effectively lead to the apoptosis of cancer cells and prevent their further growth and spread within the breast [143].

In the case of recurrent lesions, radiation therapy plays a crucial role in both local control and symptom relief [144]. Indeed, radiation therapy effectively targets the recurrent tumor, providing localized treatment and reducing the risk of spreading to other body parts. Moreover, radiation therapy can alleviate symptoms caused by recurrent lesions, such as pain, discomfort, and swelling, significantly improving the QoL for patients [145]. Treatment duration and frequency depend on the characteristics of the tumor, such as size, location, and pathology [146]. Typically, radiation therapy is delivered daily, Monday through Friday, over a span of several weeks [147]. The procedure is painless and non-invasive, but it requires precision and accuracy to ensure the maximum benefit while minimizing potential side effects.

Systemic Treatments

Systemic treatments, including chemotherapy, hormonal therapy, and targeted therapy, play a vital role in the management of primary and recurrent lesions in patients with a history of BC. These treatments aim to reduce the risk of recurrence, control the disease, and improve OS [148].

Chemotherapy is effective in cases where the cancer has spread beyond the breast or lymph nodes [149]. Chemotherapy drugs may be given intravenously or orally and lead to stopping the growth and division of cancer cells. They can help shrink tumors before surgery, eliminate any remaining cancer cells after surgery, or control the spread of the disease in advanced stages [150]. While chemotherapy can cause side effects such as hair loss, nausea, and fatigue, advancements in treatment have resulted in more targeted therapies that can minimize these effects [151].

Hormonal therapy is another systemic treatment commonly used for BC, especially in cases where the tumor is ER-positive [152]. These therapies block the effects of estrogen or reduce estrogen production. Hormonal therapy can be administered as daily pill medications or injections and is typically prescribed for a period of five to ten years; and has been shown to significantly reduce the risk of recurrence and improve OS rates [153]. Common hormonal therapy drugs include tamoxifen, aromatase inhibitors (e.g., anastrozole and letrozole), and ovarian suppression medications (e.g., goserelin) [154].

Targeted therapy is a more recent advancement in BC treatment and involves using drugs or other substances to specifically target cancer cells while minimizing damage to healthy cells [155]. For example, trastuzumab targets the HER2 protein, which is overexpressed in some BC [156]. Other targeted therapies, such as pertuzumab and lapatinib, can be combined with trastuzumab to enhance its effects [157].

## Prognostic Implications

The prognosis and treatment options for patients with BC depend on various factors, including the stage and type of the disease [158]. Additionally, the presence of primary or recurrent lesions can have prognostic implications for these patients. Larger primary lesions are often associated with a poorer prognosis, as they indicate a more advanced stage of the disease [159]. In contrast, smaller primary lesions have a better prognosis, as they may indicate an earlier stage and a higher chance of successful treatment [158][159].

Recurrent lesions often require more aggressive treatment approaches, such as targeted therapies or personalized treatment plans, to improve patient outcomes [160]. On the other hand, recurrent lesions require a different treatment approach due to their resistant nature. Additionally, recurrent lesions suggest the need for closer surveillance and more frequent follow-up visits to monitor the response to treatment and detect any further progression [161].

## Conclusion

Our study provides valuable insights into the

challenges faced in accurately distinguishing between primary and recurrent breast lesions in patients with a history of BC. Careful assessment of clinical history, physical examination findings, previous imaging studies, and molecular and genetic testing results could make an accurate diagnosis. Also, a multidisciplinary approach involving radiologists, surgeons, pathologists, and oncologists in determining the nature of suspicious lesions is necessary. However, the different diagnostic tools and techniques have some limitations and potential pitfalls. Despite the advancements in imaging technology and molecular testing, there are still challenges in distinguishing between primary and recurrent lesions. Factors such as tumor heterogeneity, treatment-related changes, and the presence of synchronous bilateral lesions further complicate the diagnostic process. Hence, further research and collaborative efforts are needed to develop more accurate and reliable diagnostic strategies, ultimately improving patient outcomes.

## Conflict of Interest

The authors declare that the research was conducted without any commercial or financial relationships that could be construed as a potential conflict of interest.
